# Roles, experiences and perspectives of the stakeholders of “10,000 Lives” smoking cessation initiative in Central Queensland: Findings from an online survey during COVID‐19 situation

**DOI:** 10.1002/hpja.598

**Published:** 2022-04-11

**Authors:** Arifuzzaman Khan, Kalie Green, Nicolas Smoll, Gulam Khandaker, Coral Gartner, Sheleigh Lawler

**Affiliations:** ^1^ School of Public Health The University of Queensland Herston Australia; ^2^ 420004 Central Queensland Public Health Unit Central Queensland Hospital and Health Service Rockhampton Australia

**Keywords:** COVID‐19, health promotion, smoking cessation, stakeholder's perspective

## Abstract

**Issue addressed:**

The “10,000 Lives” initiative was launched in Central Queensland in November 2017 to reduce daily smoking prevalence to 9.5% by 2030 by promoting available smoking cessation interventions. One of the main strategies was to identify and engage possible stakeholders (local champions for the program) from hospitals and community organisations to increase conversations about smoking cessation and referrals to Quitline. We aimed to understand the roles, experiences and perceptions of stakeholders (possible champions for delivering smoking cessation support) of the “10,000 Lives” initiative in Central Queensland, Australia.

**Methods:**

We conducted a mixed‐method online survey during the COVID‐19 situation (23 June 2020 to 22 August 2020) with a cross‐section of possible stakeholders who were targeted for involvement in “10, 000 Lives” using a structured questionnaire with mostly closed‐ended questions. Questions were asked regarding their roles, experiences and perceptions about smoking cessation and “10,000 Lives”.

**Results:**

Among the 110 respondents, 52 (47.3%) reported having provided smoking cessation support, including referral to Quitline, brief intervention and promoting existing interventions. Among them (n = 52), 31 (59.6%) were from hospitals and health services, 14 (26.9%) were from community services and three (5.8%) were from private medical practices while four of them did not report their setting. Twenty‐five respondents (22.7%) self‐identified as being directly involved with the “10, 000 Lives” initiative, which significantly predicted provision of smoking cessation support (OR 6.0, 95% CI: 2.1‐19.8). However, a substantial proportion (63.5%) of those (n = 52) who reported delivering cessation support did not identify as contributing to “10,000 Lives”.

**Conclusions:**

Stakeholders from hospitals, health services and community services are the main providers of smoking cessation support in Central Queensland. More could be done to support other stakeholders to feel confident about providing cessation support and to feel included in the initiative.

**So what?:**

Engaging with a range of stakeholders is critical for health promotion program success, to further develop the program and to ensure its sustainability. As such, funding needs to be allocated to the activities that enable this process to occur.

## 1 BACKGROUND

Globally, tobacco smoking remains one of the leading preventable causes of premature morbidity and mortality,^1^ and considerable challenges remain in ending this non‐communicable disease pandemic. While smoking prevalence has declined in many countries, gaps remain in implementing evidence‐based interventions for smoking cessation.^1^ Alongside the global tobacco epidemic,^2^ the world has been facing one of the largest communicable disease pandemics in history which has had a significant impact on people's health and health behaviours. Evidence of smoking as a risk for greater disease severity or dying from COVID‐19 is now established.^3^, ^4^, ^5^, ^6^, ^7^ Experts have recommended increasing promotion and implementation of smoking cessation interventions during this crucial time.^8^, ^9^, ^10^

The prevalence of smoking in regional and rural Australia remains considerably higher than in cities and metropolitan areas.^11^, ^12^ In Central Queensland (CQ), a regional district of Queensland, Australia located in the central east region, 16.7% of adults smoked daily in 2016 compared with the state average of 14.5%.^13^, ^14^ To address this higher smoking prevalence, Central Queensland Public Health Unit (CQPHU) launched a public health initiative called “10,000 Lives” in November 2017.^15^ The initiative's objective was to reduce daily smoking to 9.5% in CQ region by 2030 by promoting available smoking cessation interventions and advocating smoke‐free policies and programs. A detailed description of “10,000 Lives” is reported elsewhere.^15^ Briefly, the initiative employed a senior project officer (SPO) to coordinate the program's activities. The activities included promoting smoking cessation in hospitals, medical practices, educational institutes, community organisations, businesses, mining and other industries and corporate organisations, and via local radio and social media. One of the innovative and major strategies of the initiative was to identify and engage possible stakeholders (local champions for the program) from hospitals and community organisations to increase conversations about smoking cessation, referrals to smoking cessation services and overall promotion of smoking cessation in CQ.

CQ region, with a population of ~220 000, had only 61 confirmed cases of COVID‐19 (30.9 cases per 100,000 population) up to 26 December 2021.^16^ The region had only 1 week long major lockdown in April 2020 and several infrequent and minor area lockdowns (eg restriction of visits in hospitals and aged care facilities) after that. Decreased use of smoking cessation programs due to COVID‐19 has been observed in regions like Ontario, Canada (4594.3 cases per 100 000 population), where the incidence of COVID‐19 was relatively higher than in regional Australia during the first 2 years of the pandemic.^17^, ^18^ However, research suggests that motivation to quit smoking increased in countries highly affected by COVID‐19 like the United States of America and Germany in this pandemic situation.^19^, ^20^ Other research in Australia, New Zealand and the United Kingdom found that messaging about the risk of COVID‐19 among people who smoke was effective at stimulating quit intentions and behaviours.^21^ In Australia, quitting activities (eg downloading smoking cessation apps) increased substantially since the beginning of the pandemic.^9^, ^22^ However, we are unaware of any Australian study that evaluated the stakeholder's view of smoking cessation programs like “10,000 Lives” during the COVID‐19 pandemic.

We evaluated the process of “10,000 Lives” based on a standard health promotion framework and reported the inputs, activities, outputs and immediate impacts elsewhere.^15^, ^23^ We found that “10,000 Lives” successfully increased the uptake of smoking cessation interventions like Quitline by engaging stakeholders in hospitals and community services. In the first 2 years since its launch, the initiative contacted 536 stakeholders, conducted 20 educations sessions and held two summits on smoking cessation and trained nine champions to provide smoking cessation support to their clients and colleagues.^15^ Thus, “10,000 Lives” substantially increased the referrals to (by 3.8 times) and participation in (by 3.4 times) the Quitline program in the CQ region.^23^ As the campaign activities of “10,000 Lives” continued during the COVID‐19 pandemic throughout 2020, it also provided a unique opportunity to understand the stakeholder's perspective about the potential impact of the pandemic on the use of smoking cessation support in a regional area in Australia like CQ that had experienced few COVID‐19 cases at that time. Understanding the perspectives of the stakeholders who deliver and recommend smoking cessation interventions is an essential element of the process evaluation to identify potential areas for improvement and build sustainability into the program.

Therefore, we conducted a cross‐sectional survey from June to August 2020 to explore the demography and employment status of program's stakeholders, and assess their roles, experiences and perceptions about smoking cessation and “10,000 Lives”.

## 2 METHODS

### 2.1 Study design

We conducted a mixed‐method online survey among the possible stakeholders of the “10,000 Lives” initiative using a structured questionnaire with closed and open‐ended questions.

### 2.2 Study setting and population

The survey population were possible stakeholders for the “10,000 Lives” program. Possible stakeholder included employees of the hospital and health services, community services, educational institutions, business, mining or other industry, or a corporate organisation who can provide smoking cessation support to clients or colleagues, thus supporting the mission of “10,000 Lives”.^15^ Five hundred and thirty‐six possible stakeholders (among whom 171 were local champions from hospitals and community services) were identified in the first 2 years of the program by the SPO, who maintained a database of possible stakeholders for the initiative's activities (eg summits, workshops, exhibitions, meeting, phone and email communication).^15^

### 2.3 Online survey

The survey was created in the CQ Health consultation hub (https://cqhealth.citizenspace.com/) using a digital platform (Citizen Space of Delib Ltd).^24^ The survey link was emailed to the 536 possible stakeholders inviting them to complete the survey. The link was shared in the staff news bulletin of Central Queensland Hospital and Health Service (CQHHS) and staff portal email groups of council and business industries. An email reminder was sent fortnightly. The survey link was live for 2 months (23 June 2020 to 22 August 2020). The study was approved by the CQHHS Human Research Ethics Committee (HREC/2019/QCQ/50602). Participants were presented with a Participant Information Sheet outlining the study, which was approved by CQHHS Communications Department. Participants were required to confirm they had read the information and consented to participate before commencing the survey.

The survey (see Table S1) included 38 questions: three demographic questions (age, gender and locality), six about employment status including any change during COVID‐19, five about involvement with smoking cessation activities, four about the participant's role and experience with referring people to Quitline, six about the Smoking Cessation Clinical Pathway (SCCP – a standardised form to conduct a brief intervention and refer Queensland Health clients to smoking cessation support),^25^ eight that related to “10,000 Lives”, and three on COVID‐19 and smoking cessation. Most questions were multiple‐choice format including few dichotomous (eg involved with “10,000 Lives”: yes, coded as ‘1’ and no, coded as ‘0'). A five‐point Likert scale response option was used for the questions related to roles (eg smoking cessation support provided: never, rarely, sometimes, often and always), experience (usefulness of Quitline: useless, slightly useful, moderately useful, useful and very useful) and perception (how important the role of “10,000 Lives”: not important, slightly important, moderately important, important and very important). Additionally, participants were asked three open‐ended questions about resources/support needed from the initiative to perform or promote smoking cessation, and for any further comments on “10,000 Lives” or suggestions for additional action related to smoking cessation and the COVID‐19 pandemic.

### 2.4 Survey data analysis

Survey data were analysed in R version 4.0.2.^26^ Frequencies and percentages were calculated for socio‐demographics and outcome variables. The survey's primary endpoints were the categorical variables of roles, experience and perception related responses. The relationship between perceived involvement in “10,000 Lives” and experience of providing any smoking cessation support in the last year was assessed by multivariate logistic regression. In our model, the outcome variable was the experience of providing smoking cessation support (a dichotomous variable) and the independent variables were: perceived involvement in “10,000 Lives”, age group, gender and employer, each was converted to a dichotomous variable considering the most frequent response option coded as ‘1’, with all other responses option coded as ‘0’.

Responses to open‐ended questions were coded based on the interview guide to produce deductive themes (ie roles, experience, perception about smoking cessation and any comment or suggestion for “10,000 Lives”) and inductive themes, if any, by one researcher (AK) using NVivo version 12.0.^27^ We used Braun and Clarke's process for thematic analysis of the qualitative data to identify any new theme that had not been captured by the quantitative questions.^28^

## 3 RESULTS

### 3.1 Demographics of the respondents

One hundred and ten participants completed the survey. The response rate was estimated as 20.5% based on the numbers of possible stakeholders (n = 536) listed by the SPO in the first 2 years of the program launch.^15^ Of the respondents (n = 110), 83 (75.5%) were female, 61 (56%) were aged 35‐44 years, 73 (66.4%) were employed full‐time. The listed employers included a range of departments within CQHHS, community services, local government and education sector. Most respondents (n = 84; 76.4%) reported no change in their job status due to COVID‐19, yet 41.8% (n = 46) of respondents reported an increase in client interaction during the COVID‐19 period. There were no differences in most demographic and employment characteristics between the respondents who reported providing smoking cessation support and those who did not (Table 1).

**TABLE 1:** Socio‐demographics of the respondents including employment condition, and any changes after COVID‐19

Attributes	For all respondents (N = 110) Frequency (%)	Provided smoking cessation support?		*p* value^#^
		Yes (N = 52) Frequency (%)	No (N = 58) Frequency (%)	
Gender*				
Female	**83 (77.6%)**	37 (75.5%)	46 (79.3%)	
Male	24 (22.4%)	12 (24.5%)	12 (20.7%)	
Age group in year**				
18‐34	29 (26.6%)	5 (9.8%)	24 (41.4%)	**<.001**
35‐44	23 (21.1%)	14 (27.5%)	9 (15.5%)	
45‐54	**38 (34.9%)**	**25 (49.0%)**	13 (22.4%)	
55+	19 (17.4%)	7 (13.7%)	12 (20.7%)	
Current employment status				
Full time employment	**73 (66.4%)**	38 (73.1%)	35 (60.3%)	.194
Part time/casual employment	30 (27.3%)	10 (19.2%)	20 (34.5%)	
Self employment	7 (6.4%)	4 (7.7%)	3 (5.2%)	
Organisation best represent***				
Hospital and health service	59 (57.8%)	31 (64.6%)	28 (51.9%)	.150
Community services/council/education	32 (31.4%)	14 (29.2%)	18 (33.3%)	
Private medical practice	6 (5.9%)	3 (6.2%)	3 (5.6%)	
Corporate services/industries	5 (4.9%)	0 (0.0%)	5 (9.3%)	
Any change occurred with employment status due to COVID‐19?				
No change	**84 (76.4%)**	41 (78.8%)	43 (74.1%)	.562
Change	26 (23.6%)	11 (21.2%)	15 (25.9%)	
Any change occurred in your client interactions workload due to COVID‐19?				
Increased	**46 (41.8%)**	23 (44.2%)	23 (39.7%)	.809
No change	29 (26.4%)	14 (26.9%)	15 (25.9%)	
Decreased	35 (31.8%)	15 (28.8%)	20 (34.5%)	

Abbreviation: CQHHS, Central Queensland Hospital and Health Service.

* Three respondents did not answer this question,

** One respondent did not answer this question,

*** Eight respondents did not answer this question (four in second and four in third column).

^#^ *p* value is significant if <.05.

### 3.2 Roles and experience

Fifty‐two participants (47.3%) reported providing smoking cessation support (see Table 2) and 43 (82.7%) of them (n = 52) provided information on how frequently they provided the support. Nearly half (n = 21, 48.8%) of them (n = 43) reported providing this support at least once or twice a month, with over one‐third (n = 15; 34.9%) providing support several times a week. Most (n = 40; 76.9%) of the respondents (n = 52) provided support as part of their job role and/or self‐motivation (n = 16; 30.8%) and/or motivation from the Senior Project Officer (n = 10; 19.2%) (see Table 2).

**TABLE 2:** Roles and experience of the respondents (n = 52) who provided any smoking cessation support in past 1 year

Questions	For all respondents (N = 52) Frequency (%)	Employed in CQHHS (n = 31) Frequency (%)	Employed in other than CQHHS (n = 21) Frequency (%)	*p* value^#^
1. How often did you provide smoking cessation intervention?*				
Always (several times a week)	15 (34.9%)	10 (38.5%)	5 (29.4%)	.282
Sometimes (once or twice in a month)	21 (48.8%)	14 (53.8%)	7 (41.2%)	
Rarely (once or twice in a year)	7 (16.3%)	2 (7.7%)	5 (29.4%)	
2. Why did you perform smoking cessation activities?				
Part of my job role				
No	12 (23.1%)	5 (16.1%)	7 (33.3%)	.149
Yes	40 (76.9%)	26 (83.9%)	14 (66.7%)	
Self‐motivation				
No	36 (69.2%)	21 (67.7%)	15 (71.4%)	.777
Yes	16 (30.8%)	10 (32.3%)	6 (28.6%)	
Motivation from “10,000 Lives” project officer				
No	42 (80.8%)	26 (83.9%)	16 (76.2%)	.490
Yes	10 (19.2%)	5 (16.1%)	5 (23.8%)	
3. What kind of smoking cessation support did you provide?				
Were you involved in referring smokers to Quitline?				
No	10 (19.2%)	7 (22.6%)	3 (14.3%)	.456
Yes	42 (80.8%)	24 (77.4%)	18 (85.7%)	
On average, how many smokers per week did you refer to Quitline?				
Median	2.0	2.0	2.5	.587
Q1, Q3	1.0,3.0	1.0,2.0	1.0,5.0	
Did you provide brief intervention?				
No	27 (51.9%)	12 (38.7%)	15 (71.4%)	.020
Yes	25 (48.1%)	19 (61.3%)	6 (28.6%)	
Did you use Smoking Cessation Clinical Pathway (SCCP)?**				
No	16 (42.1%)	11 (39.3%)	5 (50.0%)	.556
Yes	22 (57.9%)	17 (60.7%)	5 (50.0%)	
On an average, how many SCCP did you complete per week?				
Median	4.0	3	7.0	.597
Q1, Q3	1.0, 9.2	1.0, 6.0	1.0, 10.0	
Did you promote smoking cessation intervention?				
No	30 (57.7%)	17 (54.8%)	13 (61.9%)	.613
Yes	22 (42.3%)	14 (45.2%)	8 (38.1%)	
Did you provide counselling for smoking cessation?				
No	38 (73.1%)	23 (74.2%)	15 (71.4%)	.825
Yes	14 (26.9%)	8 (25.8%)	6 (28.6%)	
Did you provide clinical treatment for smoking cessation?				
No	43 (82.7%)	26 (83.9%)	17 (81.0%)	.785
Yes	9 (17.3%)	5 (16.1%)	4 (19.0%)	

Abbreviations: CQHHS, Central Queensland Hospital and Health Service; SCCP, smoking cessation clinical pathway.

* Nine respondents did not answer this question,

** Fourteen respondents did not answer this question.

^#^ *p* value is significant if <.05.

Among the respondents (n = 52) who provided smoking cessation support, 42 (80.8%) had made about two (median: 2, IQR: 1‐3) referrals to Quitline per week in the last year. Several of them (n = 25; 48.1%) were involved in providing brief interventions and 22 (42.3%) had used the SCCP form. A substantial portion (n = 22; 42.3%) of respondents were involved in promoting smoking cessation interventions and some directly provided counselling or clinical treatment (n = 14; 26.9% and n = 9; 17.3% respectively) for smoking cessation (see Table 2).

### 3.3 Perceptions

Overall, the respondents supported the smoking cessation activities promoted by “10,000 Lives” (see Table 3). These activities included referral to the Quitline service, delivering brief interventions, and completing the SCCP for the patients who smoke. The majority of respondents (n = 75; 68.2%), especially those who work for hospital and health services (43 of 75; 72.9%), strongly acknowledged the important role of “10,000 Lives” for increasing smoking cessation in CQ. More than half (n = 63; 57.3%) of the respondents believed that people feel supported by the “10,000 Lives” initiative even during the COVID‐19 pandemic (n = 55; 50%). However, a substantial portion (n = 42; 40%) perceived that this was not always true.

**TABLE 3:** Perception of the stakeholders regarding roles of “10,000 Lives” and Quitline for smoking cessation in CQ including COVID‐19 pandemic situation

Questions	For all respondents (N = 110) Frequency (%)	Employed in CQHHS (N = 59) Frequency (%)	Employed in other than CQHHS (N = 51) Frequency (%)	*p* value^#^
Do you support/oppose smoking cessation activities in Central Queensland?				
Strongly support	**75 (68.2%)**	43 (72.9%)	32 (62.7%)	.477
Support	25 (22.7%)	12 (20.3%)	13 (25.5%)	
Neither support nor oppose	10 (9.1%)	4 (6.8%)	6 (11.8%)	
Do you support/oppose “10,000 Lives” initiative?*				
Strongly support	**67 (62.0%)**	37 (64.9%)	30 (58.8%)	.806
Support	31 (28.7%)	15 (26.3%)	16 (31.4%)	
Neither support nor oppose	10 (9.3%)	5 (8.8%)	5 (9.8%)	
How useful do you think Quitline is for helping smokers to quit smoking?**				
Very useful	25 (41.0%)	15 (44.1%)	10 (37.0%)	.809
Useful	18 (29.5%)	9 (26.5%)	9 (33.3%)	
Moderately/slightly useful	18 (29.5%)	10 (29.4%)	8 (29.6%)	
How important is the role of “10,000 Lives” for increasing smoking cessation in Central Queensland?***				
Very important	58 (59.2%)	**34 (64.2%)**	24 (53.3%)	.**022**
Important	32 (32.7%)	12 (22.6%)	**20 (44.4%)**	
Moderately/slightly important	8 (8.2%)	7 (13.2%)	1 (2.2%)	
Is it true that people in Central Queensland feel supported by “10,000 Lives” for smoking cessation?****				
Almost always true	11 (10.5%)	8 (14.5%)	3 (6.0%)	.255
Usually true	52 (49.5%)	24 (43.6%)	28 (56.0%)	
Not always true	42 (40.0%)	23 (41.8%)	19 (38.0%)	
Is it true that people feel supported by “10,000 Lives” or Quitline services for smoking cessation during the COVID‐19 situation?*****				
Almost always true	6 (6.0%)	5 (9.3%)	1 (2.2%)	.323
Usually true	49 (49.0%)	25 (46.3%)	24 (52.2%)	
Not always true	45 (45.0%)	24 (44.4%)	21 (45.7%)	
Did you notice any change in the number of people interested in quitting smoking since COVID‐19 in Central Queensland?******				
No change	68 (72.3%)	40 (76.9%)	28 (66.7%)	.152
Increased	24 (25.5%)	10 (19.2%)	14 (33.3%)	
Decreased	2 (2.1%)	2 (3.8%)	0 (0.0%)	

Abbreviation: CQHHS, Central Queensland Hospital and Health Service.

* Two respondents did not answer this question,

** Forty‐five respondents did not answer this question,

*** Twelve respondents did not answer this question,

**** Four respondents did not answer this question,

***** Ten respondents did not answer this question,

****** Sixteen respondents did not answer this question.

^#^ *p* value is significant if <.05.

Most survey participants (n = 68; 61.8%) indicated that they did not observe any changes in smoking cessation behaviour during the COVID‐19 pandemic. However, some (n = 24; 21.8%) observed an increased interest in quitting smoking. Common reasons noted included financial issues (n = 30; 27.3%) and/or changes in people's circumstances in the pandemic (eg worried about developing severe COVID‐19 and life‐threatening condition, staying at home: n = 20; 18.2%) provided a good opportunity to quit. These perceptions were comparable across the respondents from hospital and health service and other organisations. The findings related to respondent perceptions are presented in Table 3.

### 3.4 Involvement with “10,000 Lives”

We asked participants if they were involved with the “10,000 Lives” initiative as a stakeholder (local champion) for smoking cessation. Among all participants (n = 110), only 25 (22.7%) thought that they were directly involved with the “10,000 Lives” initiative. Most were female (62.5%), aged between 35–54 years (72%), from the hospital and health service (66.7%) and employed fulltime (84.0%).

For the respondents who were involved with “10,000 Lives” (n = 25), attending the summit (n = 8; 32%), meeting with the SPO (n = 4; 16.0%) and email contact (n = 4; 16.0%) were the main drivers to become involved with “10,000 Lives”. Common roles of the “10,000 Lives” champions included providing quit support to clients (n = 17; 68.0%) and/or colleagues (n = 9; 36.0%) at their workplace and/or to the people who smoke within their network (n = 8; 32.0%). Many champions (n = 15; 60.0%) promoted the activities of the initiative in the community. Table S2 demonstrates the differences in demographics and current roles of the respondents who perceived themselves involved with “10,000 Lives” and who did not. Table S3 describes their involvement and experience with “10,000 Lives”.

### 3.5 Predictors of providing smoking cessation support

**FIGURE 1:**
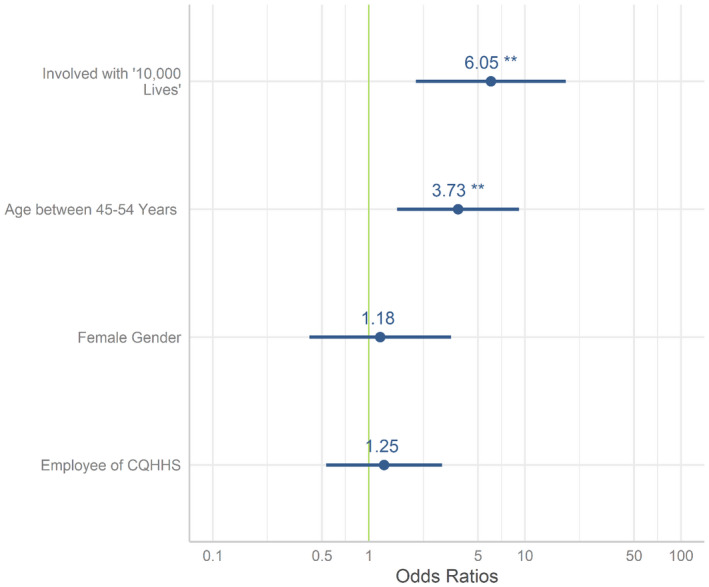
Model result indicating the predictors of providing any smoking cessation support by the stakeholders. **Indicates significant *p* < .01

Using a logistic regression model, we found that reporting involvement in “10,000 Lives” was the strongest predictor (compared with indicated they were not involved) of providing smoking cessation support in the last year; odds ratio (OR) = 6.0 (95% CI: 2.1‐19.8). We also included the demographic variables and employer of the respondent in the model and did not find any other predictors except being aged between 45 and 54 years age; OR = 3.7 (95% CI: 1.5‐9.4). Respondents aged 45‐54 years made up 35% of the full respondent list. The estimates of effect are demonstrated in Figure 1.

### 3.6 Further comments by stakeholders: Analysis of open‐ended questions

Findings from the open‐ended questions are summarised in Table 4. Three questions were asked regarding: overall comments on the activities of “10,000 Lives”, suggestions for new activities during COVID‐19 and further resources required from the initiative. We deductively identified the three themes: an overall perspective on “10,000 Lives”, further advice concerning ‘10,000’ and any resource required from “10,000 Lives”. Overall, the activities and impact of “10,000 Lives” were appraised positively by stakeholders from the different organisations. However, suggestions included enhancing the promotional activities of the initiative to raise the CQ population awareness. To do this, respondents suggested supplying promotional material that embeds information about the increased harmful effects of smoking on COVID‐19 outcomes.

**TABLE 4:** Stakeholder's insight about “10,000 Lives”: analysis of open‐ended questions data

Organisation	Summary of the insight		Representative quote		
(A) Overall comment on “10,000 Lives” activities *(Q Please add any further comments about the activities of “10,000 Lives” initiatives.)* *[*n* = 19 respondents provided any responses to this part of the question]*.					
Hospital	A great initiative with a project officer doing a good job.		*‘A great initiative. It is still difficult to embed smoking cessation in the daily practice of health care professionals so that everyone in the team is talking to the patient about smoking cessation.'…'Project officer is doing a wonderful job'*		
University	A professional and enthusiastic initiative.		*‘This is a fantastic initiative run professionally and enthusiastically. I hope it continues for the foreseeable future.'*		
Non‐Government Organisation	Impactful initiative.		*‘A truly great initiative with tangible outcomes that demonstrate its success!’*		
No organisation mentioned	Impactful initiative.		*‘It is a wonderful to support client to quit smoking. This 10,000 Lives is helping and supporting their smoking cessation goal… ’*		
(B) Any special suggestions for “10,000 Lives” during COVID *(Q Do you have any suggestions for “10,000 Lives” initiatives for doing something special in this COVID‐19 pandemic for smoking cessation?)* *[*n* = 18 respondents provided any responses to this part of the question]*.					
Hospital	Arrange more resources, education and training for the champions to provide better support			*‘Handout for smokers in emergency department about smoking and COVID‐19 and promote Quitline'…'Education throughout the healthcare system'…'Need staff training for motivational interviewing'…'More Health promotion's in the Community & Schools'…'Continue to supply the resources for quit smoking including financial support'*	
Community Health	More health promotion			*‘More Health promotion's in the Community & Schools'…'More promotional on social media especially with e‐cigarette usage.'*	
Aged care facility	Need more strict rules and regulation to enforced			*‘Until rules and regulations are strongly enforced and large fines etc and bought down smokers will still continue to be self‐centred and uncaring of the rest of the population'*	
(c) Any resource required from “10,000 Lives” *(Q Do you need any further resources/support specific to COVID and smoking?)* *[*n* = 22 respondents provided any responses to this part of the question]*.					
Hospital		Handouts embedding the information about smoking, COVID‐19 and harmful effect of smoking including morbidity and mortality data and smoking cessation. Smoking screening and quit support product and financial support			*Handout for smokers in hospital and community about smoking and COVID‐19 and promote Quitline'…'NRTs supply and CO breath monitor (smokerlyzer)'…*
Non‐Government Organisation		More frequent community outreach activities and sharing general information			*More community outreach activities supported by media such as Radio, Newspaper, Banner in Shopping centres.' …'Maybe just sharing some general information through community newsletters would be helpful, I have not seen a great deal of community'…' education/promotion regarding COVID/smoking. 'Staff training on motivational interviewing techniques to improve the quality of communications regarding smoking cessation. I have found providing non‐judgemental, patient centred conversation focusing on the persons individual reasons to quit, setting individual goals and encouraging a follow up, is more effective than asking the smoking cessation pathway questions* verbatim*.'*

We also analysed the comments inductively to identify any new themes that were not covered by deductive coding. We observed a range of attitudes towards people who smoke reflected in the comments, with only one comment indicating a negative view of people who smoke:

“Until rules and regulations are strongly enforced and large fines etc and bought down smokers will still continue to be self‐centred and uncaring of the rest of the population”.

However, other comments demonstrated a caring attitude towards people who smoke, suggesting that additional staff training in motivational interviewing could improve the ability of the champions to support people to quit smoking with greater care and empathy.

## 4 DISCUSSION

### 4.1 Interpretation of the key findings

In the current study, we sought to understand the stakeholder's roles, experiences and perceptions of the “10,000 Lives” initiative. The findings indicate that among the respondents, “10,000 Lives” had been largely successful in building a good rapport with the stakeholders from the hospitals and community, and to encourage smoking cessation activities including referrals to Quitline and brief intervention. Our previous research using an interrupted time series analysis has shown that “10,000 Lives” increased referrals to, and use of Quitline services in the CQ region.^23^ We also previously reported the process of stakeholder engagement (eg frequent contact through email, phone and face to face meeting), including the numbers of stakeholders contacted (n = 536) and the proportion (31.9%) who became champions of the initiative.^15^ The roles and experience of most of the respondents of the current survey are consistent with our previous findings. For example, consistent with the findings of our interrupted time series analysis,^23^ referral to Quitline was the most popular activity practiced by the stakeholders who were involved in providing quit support to their clients or people in their network. The important role of “10,000 Lives” for communicating and inspiring the champions to perform smoking cessation activities was acknowledged by the respondents (Tables 3 and 4). Overall, engagement with the initiative prompted them to provide support for smoking cessation.

However, many of the survey respondents perceived that they had not been directly involved with “10,000 Lives”, even though they could be possible stakeholders of the program based on their job role. According to the SPO, frequent staff turnover in hospitals and corporate organisations is associated with new staff being unaware they are a stakeholder in the program. These respondents may have not been introduced to the program by the SPO prior to the survey. Since there is only one SPO available to identify and contact all possible stakeholders in the program, new staff can remain uncontacted for some time. However, this finding could also be a reflection of stakeholder perceptions of their level of participation in the program. Interestingly, many of the stakeholders from the hospital and health service were not using the SCCP form to provide brief interventions to their clients who smoke, or refer them to Quitline, which is an expected part of their job role in providing comprehensive care. Accordingly, “10,000 Lives” needs additional strategies to increase stakeholder engagement and participation with the interventions promoted by “10,000 Lives”. A substantial number of respondents (n = 45) were not confident about the “10,000 Lives” initiative supporting people to quit smoking during this COVID‐19 pandemic situation. Also, a negative view of people who smoke demonstrated in one respondent comment may indicate the need to provide education and interventions that increase empathy towards people who smoke, because delivering smoking cessation support in an empathetic, non‐stigmatising way is crucial to help people who smoke to successfully quit.^29^, ^30^, ^31^ This information can support the “10,000 Lives” team in arranging further training and support for the stakeholders to have empathy and support of their clients to quit smoking.

A recent systematic review analysed data from a number of studies (majority from USA) that evaluated brief interventions and referral programs for substance use, where engagement of champions was found to be a key component.^32^ The review identified that good collaboration with local champions is one of the essential factors in facilitating interventions for addiction. The “10,000 Lives” initiative sought to engage champions from hospital, community and corporate services.^15^ Previous studies that evaluated smoking cessation programs from the perspective of champions/clinicians found that champions/clinicians were good sources of information on how implementation strategies of a program should be prioritised. For example, Shershneva et al^33^ reported the responses of stakeholders (clinicians engaged with the program) involved in a complex multi‐component and collaborative smoking cessation program on its reach, satisfaction level and performance outcomes, by surveying stakeholders. They found an overall positive impact of the program in reaching the stakeholders and engaging them with a variety of smoking cessation activities. Likewise, in our study we observed a positive appraisal of this locally coordinated smoking cessation initiative (ie “10,000 Lives”) from its stakeholders.

### 4.2 Limitation of the study

We acknowledge that the surveyed sample was relatively small, and response rate was at one‐fifth of the estimated total possible stakeholder number (estimated 20.5% response rate). No new themes emerged from the open‐ended questions beyond some extension of the question themes in the survey, including ongoing challenges to the sustainability of the “10,000 Lives” program. A time‐constrained environment is one of the biggest challenges in a clinical setting however this can be overcome by developing a clear strategy and implementing a standardised process.^34^ For example, the “10,000 Lives” initiative intensively promotes the SCCP^25^ tool to support clinicians in routinely identifying patients who smoke and in delivering a brief intervention.

### 4.3 Implications and recommendations

Our study provides important recommendations for the “10,000 Lives” initiative and health promotion programs more generally. The initiative should find ways to increase engagement with possible stakeholders to ensure those who are yet to be involved are encouraged to participate. This could be done by employing additional program staff to contact and engage stakeholders more frequently.

Engaging with the range of possible stakeholders that have interests in promoting health interventions is critical for its success, and to support iterative development of the program. As such, funding needs to be allocated to the activities that enable this process to occur. A regional smoking cessation initiative like “10,000 Lives” can be replicated by public health services in other geographic areas with appropriate local tailoring to increase smoking cessation activities in the region.

## 5 CONCLUSION

A diverse range of stakeholders of the “10,000 Lives” were identified from the survey. Many of them participated in the program and provided smoking cessation support to their clients or colleagues who smoke. The perception of participation and engagement with the initiative among the respondents varied. Stakeholders acknowledged the important role of “10,000 Lives,’ however strategies are needed to increase participation, and confidence in working with people who smoke, to ensure sustainability of the initiative. Stakeholders are integral to the success of health promotion programs (eg smoking cessation initiatives) particularly in regional areas where resources are tight. Lessons learnt from this study should inform others to identify appropriate and realistic inputs and activities for the design and implementation of health promotion programs.

## CONFLICT OF INTEREST

None declared.

## ETHICS APPROVAL STATEMENT

Ethics approval were obtained for the study from Central Queensland Hospital and Health Service Human Research Ethics Committee (HREC) (Approval no: HREC/2019/QCQ/50602) and was ratified by the HREC of the University of Queensland (Clearance Number: 2019001760).

## AUTHOR CONTRIBUTIONS

AK conceptualised the study and wrote the first draft. CG and SL contributed to the development of the concept. AK designed the questionnaire after discussion with KG, NS, GK, CG and SL. AK performed the primary analysis and interpretation of the data. AK, KG, NS, GK, SL and CG critically reviewed the data analysis and interpretation. All authors contributed with critical revisions to the content of the manuscript. The final version of the manuscript was approved by all authors.

## Supporting information

Table S1

Table S2‐S3
