# Supplementary material for: Roles, experiences and perspectives of the stakeholders of “10,000 Lives” smoking cessation initiative in Central Queensland: Findings from an online survey during COVID‐19 situation
Source: Health Promot J Austr. 2022 Apr 11:10.1002/hpja.598. Online ahead of print. doi: 10.1002/hpja.598 (PMC9087515; doi:10.1002/hpja.598)
Supplement: Supplementary file 1 — Table S1 [file HPJA-9999-0-s002.docx]

**SUPPLEMENTARY TABLE S1**

# **Role, experience and perception about “10,000 Lives” and smoking cessation activities in Central Queensland**

## **OVERVIEW**

Currently, we are facing one of the largest pandemics of history which has significant consequences on people’s health and wellbeing. Community lockdowns and self-isolation may impact on mental health and lead to changes in some behaviours such as tobacco smoking. Consequently, you may notice a change in the interests for smoking cessation by the people who smoke. In this context, we seek to understand your role, experience and perception about “10,000 Lives” and smoking cessation activities in CQ and invite you to participate in an online survey.

Please read the 'Participant Information Sheet' attached below. You can get the detail

information about "10,000 Lives" through the below link.

<https://cqhealth.citizenspace.com/central-queensland-public-health-unit/10000lives-smokingcessation-covid-19>

## **CONSENTING**

1. **I read and understand the purpose, risk, benefit and confidentiality issues of participating in the survey from the survey information sheet.**

- Yes
- No => Please review the survey detail again.

1. **I voluntarily agree to participate in the survey**

- Yes
- No => Please stop here

## **SURVEY QUESTIONNAIRE**

### **Section A: Your demographics**

1. **What is your age in years?**

____________

1. **What is your Gender?**

- Female
- Male
- Other
- Prefer not to say

1. **What is the postcode of your residence?**

____________

### **Section B: Employment status**

1. **What is your current employment status?**

*Please select the response which best answers this question:*

- Employed (full time)
- Employed (part time)
- Self-employed => skip to Section D
- Not currently employed => skip to Section D

### **Section C: Your work information**

1. **Postcode of your current workplace address?**

____________________

1. **Please tell us which organisation you best represent**

*Please select the response which best answers this question:*

- Hospital
- Community Health
- Community Service
- GP
- Council
- Educational setting (e.g. School, TAFE)
- Non-Government Organisation
- Voluntary organisation
- University
- Other smoking cessation initiatives/program
- Other: Specify______________

### **Section D: Employment Status Changed due to COVID-19?**

1. **Changes occurred on your employment status for the impact of COVID-19?**

*Please select the response which best answers this question:*

- Lost my job
- Moved to lower level position
- Lost hours/shift
- No change
- Gained hours/shift
- Promoted to upper level position
- Got a new job

1. **Any changes occurred to your workload due to COVID-19?**

*Please select the response which best answers this question:*

- Markedly/Greatly Decreased
- Decreased
- No Change
- Increased
- Markedly/Greatly Increased

1. **Client interactions changed due to the impact of COVID-19 pandemic?**

- Markedly Decreased
- Decreased
- No Change
- Increased
- Markedly Increased

### **Section E: Smoking cessation activities in Central Queensland**

1. **Do you support/oppose smoking cessation activities in Central Queensland?**

*Please select the response which best answers this question:*

- Strongly Oppose
- Oppose
- Neither Support nor Oppose
- Support
- Strongly Support

1. **Are/were you involved with any smoking cessation activities?**

- Yes
- No => skip to Section G

### **Section F: Your role in smoking cessation**

1. **What kind of smoking cessation activities did you deliver?**

*Please select all the responses that apply:*

- Referring smokers to Quitline
- Brief intervention
- Counselling
- Clinical treatment for smoking cessation
- Health promotion for smoking cessation
- Run smoking cessation program
- Provide training on smoking cessation
- Other: Specify______________

1. **How often did you perform smoking cessation activities in last one year?**

*Please select the response which best answers this question:*

- Never
- Rarely (once or twice in a year)
- Sometimes (once or twice in a month)
- Often (once a week)
- Always (several times a week)

1. **Why did you perform smoking cessation activities?**

*Please select all the responses that apply:*

- Part of my job role
- Involvement with “10,000 Lives”
- Involvement with a smoking cessation initiative/program other than “10,000 Lives”
- Self-motivation
- Motivation by the senior project officer of “10,000 Lives”
- Motivation from social media
- Motivation from Other media
- Other: Specify______________

### **Section G: Quitline**

*Quitline is a one-stop service for the people who want to quit smoking. The service includes telephone counselling and Nicotine replacement therapies. Since February 2017, all Central Queensland smokers are eligible for:*

- *12 weeks of free nicotine replacement therapy (posted to smoker's home)*
- *4 confidential call backs with a Quitline counsellor (free)*

1. **Do you support/oppose Quitline services for smoking cessation in Central Queensland?***Please select the response which best answers this question:*

- Strongly Oppose
- Oppose
- Neither Support nor Oppose
- Support
- Strongly Support

1. **Are/were you involved in referring smokers to Quitline?**

- Yes
- No => Skip to Section I

### **Section H: Experience and perspective about Quitline**

1. **On average, how many smokers per week did you refer to Quitline from Central Queensland in the last one year?**

*Please put the estimated numbers of smokers you referred weekly in your past 1-year experience*

__________________

1. **How useful do you think Quitline is for helping smokers to quit smoking?**

- Useless
- Slightly Useful
- Moderately Useful
- Useful
- Very Useful

### **Section I: Smoking Cessation Clinical Pathway (SCCP)**

1. **Do you know about Smoking Cessation Clinical Pathway form?***Please select the response which best answers this question:*

- Yes
- No Skip to Section M

### **Section J: Used Cessation Clinical Pathway?**

1. **Do/did you use** **Smoking Cessation Clinical Pathway form in the last one year?***Please select the response which best answers this question:*

- Yes
- No Skip to Section L

### **Section K: Experience and perspective about Smoking Cessation Clinical Pathway**

1. **On average, how many Smoking Cessation Clinical Pathway forms do you complete per week?**

*Please put in the estimated numbers of SCCP forms per week did you fill out in your past 1-year experience, here:*

*__________________*

1. **How useful is the Smoking Cessation Clinical Pathway in having a conversation about smoking cessation?**

- Useless
- Slightly Useful
- Moderately Useful
- Useful
- Very Useful

**=>skip to section L**

### **Section L: Question on why not Smoking Cessation Clinical Pathway?**

1. **Why did you not use Smoking Cessation Clinical Pathway tool?**

*Please select all responses that apply*

- I don’t find this tool useful
- This is not my role
- I don’t have enough time to use this
- My clients are not interested to it
- I don’t feel confident to use this tool
- I don’t want to upset my clients
- Other: Specify____________________________

### **Section M: “10,000 Lives”**

*“10,000 Lives” is a smoking cessation initiative of Central Queensland Hospital and Health Service, coordinated by Central Queensland Public Health Unit which was officially launched on 1 November 2017.*

*More detail:* [*https://clinicalexcellence.qld.gov.au/improvement-exchange/10000-lives-cq-smoking-cessation-project*](https://clinicalexcellence.qld.gov.au/improvement-exchange/10000-lives-cq-smoking-cessation-project)

1. **Do you support/oppose “10,000 Lives” smoking cessation initiative in Central Queensland?**

*Please select the response which best answers this question:*

- Strongly Oppose
- Oppose
- Neither Support nor Oppose
- Support
- Strongly Support

1. **Are/were you involved with “10,000 Lives” initiative?**

- Yes
- No => skip to section O

### **Section N: Your involvement and role in “10,000 Lives”**

1. **How did your involvement in “10,000 Lives” begin?**

*Please select the response which best answers this question*

- After attending a summit/workshop
- After getting email from senior project officer
- After getting phone call from senior project officer
- After face to face meeting with senior project officer
- After seeing post in Facebook
- After seeing information in staff newsletter
- After hearing information in local radio
- After seeing article in website
- Others Specify___________________

1. **What is/was your role with “10,000 Lives” initiative?**

*Please select all the response that apply*

- Provide support for smoking cessation to my client/s who smoke
- Provide support for smoking cessation to my colleague/s who smoke
- Provide support for smoking cessation to people in my network who smoke
- Refer people who smoke to Quitline
- Promote the activities of “10,000 Lives”
- Provide training to other people on smoking cessation support
- Act as a point of contact of my organisation for “10,000 Lives”
- Other: Specify________________________________

1. **How frequently did you perform smoking cessation activities before the launch of “10,000 Lives” initiative on November 2017 in Central Queensland?**

*Please select the response which best answers this question*

- Never
- Rarely (once or twice in a year)
- Sometimes (once or twice in a month)
- Often (once a week)
- Always (several times a week)

1. **How frequently did you perform smoking cessation activities after the launch of “10,000 Lives” initiative on November 2017 in Central Queensland?**

*Please select the response which best answers this question*

- Never
- Rarely (once or twice in a year)
- Sometimes (once or twice in a month)
- Often (once a week)
- Always (several times a week)

### **Section O: Perception about “10,000 Lives”**

1. **How important is the role of “10,000 Lives” for increasing smoking cessation in Central Queensland?**

*Please select the response which best answers this question:*

- Not important
- Slightly Important
- Moderately Important
- Important
- Very Important

1. **Is it true that people in Central Queensland feel supported by “10,000 Lives” for smoking cessation?**

*Please select the response which best answers this question*

- Almost Never True
- Usually Not True
- Occasionally True
- Usually True
- Almost Always True

### **Section P: COVID-19 and smoking cessation**

1. **Did you notice any change in the number of people interested in quitting smoking since COVID-19 in Central Queensland?**

*Please select the response which best answers this question:*

- Markedly Decreased
- Decreased
- No Change
- Increased
- Markedly Increased

1. **If you observed increases in interest for smoking cessation since COVID-19 what do you think are the reasons?**

*Please select all responses that apply*

- No; interest for quitting smoking has not been increased
- People have experienced a change in circumstances, and think it is an opportunity to quit
- Financial issues
- Scared of getting COVID-19
- Scared of life-threatening condition from COVID-19
- Staying at home more and don’t smoke at home
- Other: Specify____________________________

1. **Is it true that people feel supported by “10,000 Lives” or Quitline services for smoking cessation during the COVID-19 pandemic in Central Queensland?** *Please select the response which best answers this question:*

- Almost Never True
- Usually Not True
- Occasionally True
- Usually True
- Almost Always True

### **Section Q: Advice and comments for “10,000 Lives”**

1. **Do you need any further resources/support specific to COVID and smoking?**

**If so, what would you need?**

Please answer in your own words

­­­­­­­­­­­­­­­­­­­­­­__________________________________________________________

1. **Please add any further comments about the activities of “10,000 Lives” initiatives?**

Please write in your own words

__________________________________________________________

1. **Do you have any suggestions for “10,000 Lives” initiatives for doing something special in this COVID-19 pandemic for smoking cessation?**

Please answer in your own words

__________________________________________________________

**We may conduct in-depth interviews in the future, if you would be willing to be contacted about the study, please provide your email (which will not be linked to any of the answers above)**

1. 🞏 Please check the box if you agree for this and provide your email address:
2. **Email address:** _____________________

**Thank you so much for your time.**
