# Supplementary material for: Roles, experiences and perspectives of the stakeholders of “10,000 Lives” smoking cessation initiative in Central Queensland: Findings from an online survey during COVID‐19 situation
Source: Health Promot J Austr. 2022 Apr 11:10.1002/hpja.598. Online ahead of print. doi: 10.1002/hpja.598 (PMC9087515; doi:10.1002/hpja.598)
Supplement: Supplementary file 2 — Table S2‐S3 [file HPJA-9999-0-s001.docx]

**SUPPLEMENTARY TABLES S2 and S3**

**S2: Differences in demographics and current roles of the respondents who perceived they are involved with “10,000 Lives” and who are not.**

| **Characteristics** | ***Are you involved with "10,000 Lives"?*** | | |
| --- | --- | --- | --- |
|  | **Yes (N=25)**  **Frequency (%)** | ***No (N=85)***  **Frequency (%)** | ***p value^#^*** |
| ***Gender*** |  |  | **0.044** |
| - Female | **15 (62.5%)** | 68 (81.9%) |  |
| - Male | 9 (37.5%) | 15 (18.1%) |  |
| *- Missing values* | *1* | *2* |  |
|  |  |  |  |
| ***Age group in year*** |  |  | **0.042** |
| - 18-34 | 3 (12.0%) | 26 (31.0%) |  |
| - 35-44 | **10 (40.0%)** | 13 (15.5%) |  |
| - 45-54 | 8 (32.0%) | 30 (35.7%) |  |
| - 55+ | 4 (16.0%) | 15 (17.9%) |  |
| - *Missing values* | *0* | *1* |  |
|  |  |  |  |
| ***Organisation best represent*** |  |  | 0.539 |
|  |  |  |  |
| - Hospital and Health Service | **16 (66.7%)** | 43 (55.1%) |  |
| - Community Services/Council/Education | 7 (29.2%) | 25 (32.1%) |  |
| - Private Medical Practice | 1 (4.2%) | 5 (6.4%) |  |
| - Corporate services/industries | 0 (0.0%) | 5 (6.4%) |  |
| - *Missing values* | *1* | *7* |  |
|  |  |  |  |
| ***Current employment status*** |  |  | 0.102 |
| - Full time employment | **21 (84.0%)** | 52 (61.2%) |  |
| - Part time/casual employment | 3 (12.0%) | 27 (31.8%) |  |
| - Self employment | 1 (4.0%) | 6 (7.1%) |  |
| Notes: *^#^* *p value is significant if <0.05* | | | |

**S3. Demonstrating how the stakeholders (n=25) involved, what was their role with the “10,000 Lives” initiative and their experiences before and after the launching of the initiative**

| **Questions** | ***Total respondents (N=25)***  ***Frequency (%)*** | ***Employed in CQHHS (N=16)***  ***Frequency (%)*** | ***Employed in Other than CQHHS (N=9)***  ***Frequency (%)*** | ***p value*** |
| --- | --- | --- | --- | --- |
| ***How did your involvement in “10,000 Lives” begin?*** | | | | 0.283 |
| - After attending a summit/workshop | 8 (32.0%) | 6 (37.5%) | 2 (22.2%) |  |
| - After face to face meeting with senior project officer | 4 (16.0%) | 2 (12.5%) | 2 (22.2%) |  |
| - After getting email from senior project officer | 4 (16.0%) | 1 (6.2%) | 3 (33.3%) |  |
| - After getting phone call from senior project officer | 1 (4.0%) | 1 (6.2%) | 0 (0.0%) |  |
| - After seeing article in website | 2 (8.0%) | 0 (0.0%) | 2 (22.2%) |  |
| - After seeing information in staff newsletter | 2 (8.0%) | 2 (12.5%) | 0 (0.0%) |  |
| - Becoming a staff member within a youth organisation | 1 (4.0%) | 1 (6.2%) | 0 (0.0%) |  |
| - Part of initial working party. | 1 (4.0%) | 1 (6.2%) | 0 (0.0%) |  |
| - SCCP conversations as a part of daily tasks in my new role | 1 (4.0%) | 1 (6.2%) | 0 (0.0%) |  |
| - Wrote the first draft of the proposal to run the program | 1 (4.0%) | 1 (6.2%) | 0 (0.0%) |  |
| ***What is/was your role with “10,000 Lives” initiative?*** | | | | 0.915 |
| ‐    Provide support for smoking cessation to my client/s who smoke | 17 (68.0%) | 11 (68.8%) | 6 (66.7%) |  |
| ‐    Refer people who smoke to Quitline | 16 (64.0%) | 11 (68.8%) | 5 (55.6%) |  |
| ‐    Promote the activities of “10,000 Lives” | 15 (60.0%) | 11 (68.8%) | 4 (44.4%) |  |
| ‐    Provide support for smoking cessation to my colleague/s who smoke | 9 (36.0%) | 5 (31.2%) | 4 (44.4%) |  |
| ‐    Provide support for smoking cessation to people in my network who smoke | 8 (32.0%) | 5 (31.2%) | 3 (33.3%) |  |
| ‐    Act as a point of contact for my organisation for “10,000 Lives” | 4 (16.0%) | 2 (12.5%) | 2 (22.2%) |  |
| Provide training to other people on smoking cessation support | 3 (12.0%) | 2 (12.5%) | 1 (11.1%) |  |
| ***How frequently did you perform smoking cessation activities before the launch of “10,000 Lives”?*** | | | | 0.17 |
| - Always (several times a week) | 1 (4.0%) | 1 (6.2%) | 0 (0.0%) |  |
| - Often (once a week) | 4 (16.0%) | 2 (12.5%) | 2 (22.2%) |  |
| - Sometimes (once or twice in a month) | 10 (40.0%) | 9 (56.2%) | 1 (11.1%) |  |
| - Rarely (once or twice in a year) | 7 (28.0%) | 3 (18.8%) | 4 (44.4%) |  |
| - Never | 3 (12.0%) | 1 (6.2%) | 2 (22.2%) |  |
| ***How frequently did you perform smoking cessation activities after the launch of “10,000 Lives”?*** | | | | 0.423 |
| - Always (several times a week) | 4 (16.0%) | 2 (12.5%) | 2 (22.2%) |  |
| - Often (once a week) | 3 (12.0%) | 3 (18.8%) | 0 (0.0%) |  |
| - Sometimes (once or twice in a month) | 12 (48.0%) | 8 (50.0%) | 4 (44.4%) |  |
| - Rarely (once or twice in a year) | 5 (20.0%) | 2 (12.5%) | 3 (33.3%) |  |
| - Never | 1 (4.0%) | 1 (6.2%) | 0 (0.0%) |  |
| ***Abbreviations:*** *CQHHS- Central Queensland Hospital and Health Service*  *^#^* *p value is significant if <0.05* | | | | |
